# Observation of the unexpected morphology of graphene wrinkle on copper substrate

**DOI:** 10.1038/s41598-017-08159-8

**Published:** 2017-08-15

**Authors:** Wen Wang, Shudu Yang, Ashu Wang

**Affiliations:** 1 School of Mechanical Engineering, Southwest Jiaotong University, Chengdu, 610031 China; 2 Sichuan Branch of Meteorological Training Institute CMA, Chengdu, 610072 China; 3 School of Information Science and Engineering, Chengdu University, Chengdu, 610106 China

## Abstract

Graphene, a two-dimensional material, has a wide range of unique properties and could be used in the development of varieties of mechanic, electronic and photonic devices, therefore methods to synthesis large-area high-quality graphene films are urgently required. Chemical vapor deposition (CVD) has been of particular interest recently due to its simplicity and low cost. However, because of the mismatch of thermal expansion coefficients, high densities of wrinkles are commonly observed. Despite their prevalence and potential impact on large-scale graphene properties, relatively little is known about their structural morphology and formation mechanism. In this article, morphologies of graphene obtained by CVD are experimentally investigated by an atomic force microscope (AFM) and results show that the profiles of wrinkles are much larger than they should be. By using theoretical methods and molecular dynamics simulations (MD), we find internal molecules created during CVD process which supply additional pressure is the main mechanism.

## Introduction

Graphene as a two-dimensional (2D) material has many unique physical and mechanical properties, including highest strength, elastic stiffness, in-plane electrical conductivity and thermal conductivity^1–5^. Graphene is widely supposed to be a material to enable new development of many modern technologies^4, 6^ which involves the potential applications in mechanic, electronic and photonic devices^7–10^. However, wrinkling is a ubiquitous phenomenon in 2D membranes that severely weaken the performance of graphene. In spite of very fast improving methods on manufacturing graphene, a large-area high-quality wrinkle-free graphene has yet to emerge. The practical difficulty to use graphene in devices is that the available methods for growing graphene contain high density of wrinkles^11^. Systematically understand the mechanism of wrinkle formation can help controlling its morphology and further eliminating wrinkles.

Due to its low cost and efficiency, CVD has been widely adopted to manufacture graphene. A particular issue for graphene obtained by CVD is the presence of high density of wrinkles. These are formed by differential thermal expansions, as the metal contracts more than the graphene during cooling process, leaving an excess area of graphene^12–15^. Despite their prevalence and potential impact on large-scale graphene properties, relatively little is known about their structural morphology and formation mechanism^16, 17^. In this letter, we use AFM to characterize surface morphology of graphene wrinkles and find morphology of graphene wrinkles measured by AFM is not real. The widths of wrinkle are much wider than theory and simulation results that cannot be understood. By using MD simulations and theoretical methods, we quantitatively studied the unexpected mechanism of wrinkles.

## Results

### Experiment details

**Figure 1:**
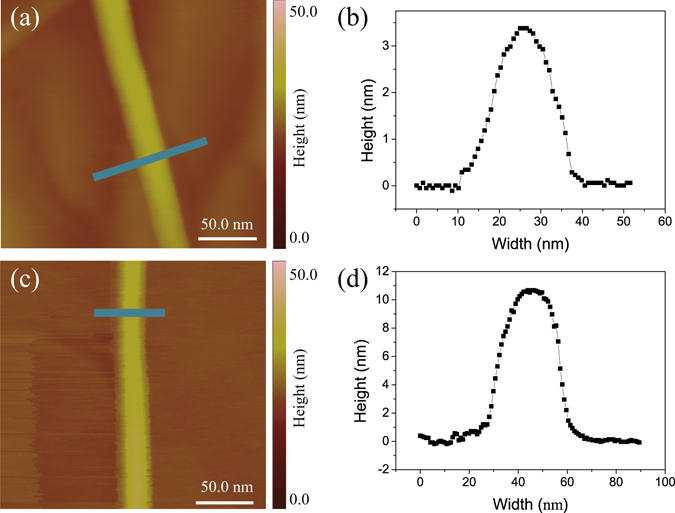
Experimental AFM morphology image of graphene wrinkles on copper sustrate by using super-sharp and high resolution tips. (**a**,**c**) AFM morphology image of graphene wrinkles obtained by CVD methods. (**b**,**d**) The AFM heights of the cross-sectionl trace of the line marked in Fig. 1a and c respectively.

Single layer graphene samples were obtained by CVD methods^18^ which were grew on copper foil substrate at temperatures up to 1000 °C. Generally copper foil has a positive thermal expansion coefficient of 16.5 × 10^−6^/K, however, graphene has an negative in-plane thermal expansion coefficient of −8 × 10^−6^/K^19^. This contrast leads to a compression strain about 2.45% between graphene films and copper substrate after reduced to room temperature. The binding between graphene and copper is weak and graphene can slip on copper surface^20, 21^, as a consequence the strain distribution is no longer uniform, typically, wrinkles can be easily found in large strain areas. In order to obtain more quantitative morphological information about wrinkles, AFM in tapping mode was adopted to investigate profiles of wrinkles. We first used conventional AFM tips with the typical diameter of ~10 nm in tapping mode to measure heights and widths of graphene wrinkle. We measured 10 graphene wrinkles, the typical measured profiles can be found in Fig. S1. Experimental measurement results show that widths of graphene wrinkles are in the range of tens of nanometers and with heights in the range of a few nanometers. Considering the fact that the width of wrinkles and the radius of tips are in the same order, shapes of AFM tips may have effect on the accuracy of wrinkle’s morphologies^22^. To avoid the effect and obtain more accurate profiles of graphene wrinkles, high resolution super sharp tips (Hi’Res-C14/Cr-AU from Micromasch) with tip spike radius of ~1 nm and spike height 100–200 nm are used in the paper. Figure 1a and c show the AFM morphological images of typical graphene wrinkles scanned in tapping mode. Total 8 graphene wrinkle were measured. The AFM heights of the the cross-sectionl trace of the line marked in Fig. 1a and c are shown in Fig. 1b and d respectively. Experimental measurements clearly show that widths of graphene wrinkles are 30 nm ≤ 2 *W* ≤ 40 nm, heights of graphene wrinkles are 3.4 nm ≤ *h* ≤ 11 nm. The profiles of wrinkles in the article are consistent with the previous results^16^ but the unexpected morphologies and mechanisms are not clear.

### Theory analysis without interlayer molecules

The binding energy (Γ)^23^ between graphene foil and copper substrate can be used to verify the accuracy of the measured morphology. We do this in the framework of the elastic theory, generalized to account for bending energy (*E*_b_) and adhesion energy (*E*_a_). In this model, the total energy consists of two parts: the bending energy stored in the graphene and the adhesion energy that represents the atomic interactions (binding) between graphene and copper substrate. The only unknown inputs to the model are the height and width of wrinkles.

According to the measured results, profiles of wrinkles can be reasonably assumed to satisfy ${y}_{s}=\frac{1}{2}h(1+\,\cos (\frac{\pi x}{W}))$ and *E*_b_ can be simply written as ${E}_{b}=\frac{Db}{2}{\int }_{-W/2}^{W/2}{({{y}_{s}}^{{\prime\prime} })}^{2}{\rm{d}}x=\frac{Db{h}^{2}{\pi }^{4}}{16{W}^{3}}$, where *D* = 1.2 eV = 0.192 nN·nm^24^ is the bending stiffness graphene, *b* represents length of wrinkle along its axis, *h* is height of wrinkles and *W* is half width of wrinkles. The adhesion energy (*E*_a_) between graphene and copper can be calculated as *E*_*a*_ = *b*Γ*W*, where Γ represents the binding energy between graphene and copper per unit area. In the equilibrium state *E*_*b*_−*E*_*a*_ = 0. By using the experimental measurements of *h* and *W*, the obtained adhesion energy Γ ≤ 0.0034 J/m^2^. Recently, Γ has been determined by experiments and first principle calculations. The reported value 0.397 J/m^2^ ≤Γ ≤ 0.72 J/m^2^ ^20, 21^, which is at least 2 orders of magnitude large than the results we estimated. These observations, taken together, clearly demonstrate that the profiles of graphene wrinkles measured by AFM are not real, however, little is known about the factors determining the profiles of wrinkles so far. Morphologies may depend on the process of CVD growth and environment; however, previous studies haven’t already yielded insight into the mechanism.

**Figure 2:**
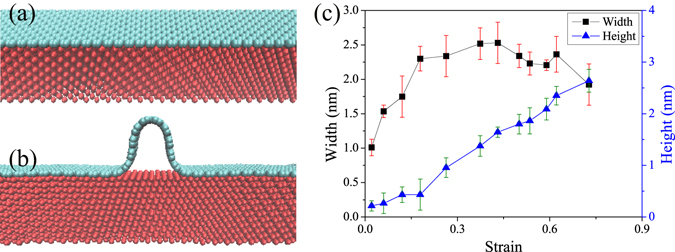
MD simulation models and calculated results, *T* = 300 K. (**a**) The MD simulation model is a stack of two rectangular materials, upper cyan graphene and lower red copper. (**b**) After the pre-compression being released, a typical graphene wrinkle is clearly formed on copper substrate. (**c**) Simulation results of graphene wrinkles vesus strain, for small strain, both *h* and *W* increase with strain; for large strain, *h* still increases with strain, however, *W* surprisingly decreses with strain. The error bars represent the standard deviation of five independent calculations.

To better understand the unexpected morphology of graphene wrinkles, we performed several MD simulations to help understanding the machanism. The model itself is a stack of a rectangular copper substrate (red part in Fig. 2a) and a single layer graphene (cyan part in Fig. 2a), with the basal planes of both parallel. In order to obtain wrinkles, a pre-compressive strain was applied to graphene layer. Then a horizontal spring with stiffnes *k* = 100 eV/Å^2^ was applied to each end of graphene in order to fix the both ends. Finally the whole model were relaxed under a constant tempereture *T* = 300 K, as a result, many wrinkles with variety of profiles were formed on copper substrate.

**Figure 3:**
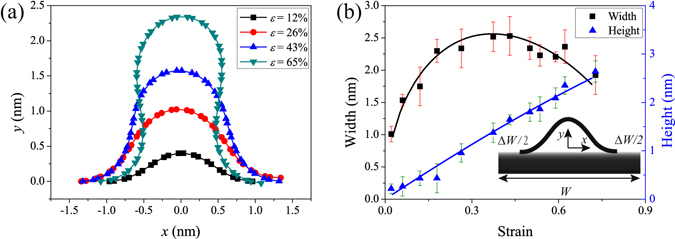
Typical cross-sectional shape of graphene wrinkles under different strain obtained by MD simulation (*T* = 300 K) and the comparison between theory and simulation results. (**a**) Typical cross-sectional shape of graphene wrinkles under strain form 12% to 65%. (**b**) Comparison to the simulation results, the theory fit and predict the results well. The error bars represent the standard deviation of five independent calculations for the same model.

Wrinkles are formed by differential thermal expansions, as the metal substrate contracts more than the graphene during cooling process. So it should form uniform random distribution wrinkles, however, the binding between graphene and copper is weak and graphene can slip on copper surface, as a consequence the strain distribution is no longer uniform which leads to non-uniform distribution of wrinkles. This phenomenon was confirmed by scanning electron microscopy images in supplementary Fig. S2(a). We observed that neighbored wrinkles can merge to form larger wrinkles in the MD simulation, see supplementary Fig. S2(b)–(d). There is a large strain of graphene in the area containing wrinkles and small strain in the area without wrinkles. What’s more, the copper substrates used in CVD growth are polycrystalline with various thickness, defects and crystal orientations which leads to very large local strain. Base on the upper phenomenon, we can conclude that wrinkles are formed by two processes. At first, high density of small wrinkles with ripple structure are formed due to thermal compression strain. Second, if there are no pining between graphene and copper substrate, neighbored wrinkles merged to form wrinkles with large strain mushroom structure and thus decrease density of wrinkles. In the MD calculations, strain is defined as *ε* = Δ*W/W* (see insert of Fig. 3b), where *W* is the flat graphene length without forming wrinkle and Δ*W* represents the difference of wrinkle’s width and length of flat graphene.

Because of the merge of wrinkles and Fig. 2b shows a typical obtained graphene wrinkle on copper with strain *ɛ* = 17.7%. Here we systematicaly invetigated morphology of graphene wrinkles under different strains (*T* = 300 K, 12.4% < *ɛ* < 68.0%) using MD simulations. *h* (blue triangle) and *W* (black rectangle) are plotted in Fig. 2c as a function of *ɛ*. From these results, we see that *h* is nearly linear dependent on strain over the entire strain range examined. On the other hand, *W* first increases with strain and then decreases with the further increase of strain. The maximun value of *W* is less than 2.7 nm. To understand the evolution of wrinkle, we ploted the typical cross-sectional profiles in Fig. 3a. Examination of Fig. 3a clearly demonstrates the evolution of graphene wrinkles. In general, the evolution of wrinkles can be divided into three part: (I) For small strain, the cross-cectional of wrinkles has a ripple shape and both *h* and *W* increase with strain; (II) The cross-section of wrinkles transited from arch shape to mushroom shape, at this time, *h* still increases with increases strain, however, *W* decreses with strain; (III) With further increasing strain, wrinkles of graphene collapsed due to the fluctuation of temperature or other influence, such as mechanical loads, electrostatic interaction and environmental disturbance. The simulation results are contradict with the experimental observes in the letter and previous reports^16^.

The profiles of wrinkles can be easily understood at a qualitative level, as arising from the competition between elastic bending energy and van der Waals binding energy^25^. At the stage of ripple structure, the bending energy is equal to the binding energy, resulting in a stable structure. As the strain increase, unstable mushroom shape structure are formed. For a given mushroom shape structure, the binding energy increases with length, while the bending energy is almost constant, mostly proportional to the number of sharp bends. When beyond the critical strain, unstable mushroom shape structure collapses to form mulilayer folds due to the fluctuation of temperature or other disturbances such as mechanical loads, electrostatic interaction and environmental disturbance in order to reduce the free energy of system^26^.

Temperature effects on profiles of wrinkles are also investigated in the article. MD culculations carried at *T* = 1 K are plotted in supplementary Fig. S3. One can find temperature almost has no effect (within calculation errors) on profiles of graphene wrinkles due to the binding energy and bending stiffness changes little with temperature.

To estimate the maximum width of wrinkles, we turn to theoretical analysis. The insert of Fig. 3b demonstrates the theoretical model. In order to account the effect of interface binding energy, Lagrange multiplier method was adopted for finding the local minima of total energy. Using fixed boundary conditions at both ends of graphene wrinkles, the total energy (*U*) can be written as follows

1 $$U(\theta ,s,\alpha )={\int }_{-W/2}^{W/2}(\frac{1}{2}D{(\frac{\partial \theta }{\partial s})}^{2}-{\rm{\Gamma }}){\rm{d}}s-\alpha (W-{\rm{\Delta }}W-{\int }_{-W/2}^{W/2}\cos \,\theta {\rm{d}}s)$$

where *D* represents the bending stiffness of single layer graphene, *α* is a Lagrangian multiplier. The first term represents the total free energy; the second term is the boundary conditions.

Taking account of fixed boundary conditions, i.e. at the end of wrinkles $s=\pm \frac{W}{2}$, *θ* = 0, cos*θ* = 1 and calculate the gradient of Eq. (1) ∇_*θ*,*s*_ *U*(*θ*, s, *α*) = 0. Finally one can obtain the following relationship

2 $$\frac{\partial \theta }{\partial s}=\sqrt{\frac{2{\rm{\Gamma }}}{D}\frac{(\cos \,\theta \,-\,\cos \,{\theta }_{0})}{(1-\,\cos \,{\theta }_{0})}}$$

*θ* = *θ*_0_ is the inflection point of wrinkles at which the curve changes from being concave (concave downward) to convex (concave upward), or vice versa, obviously $\frac{\partial \theta }{\partial s}(\theta ={\theta }_{0})=0$.

Using Eq. (2), the excess length between graphene and copper substrate (Δ*W*), the width of wrinkle (*W* − Δ*W*), the height of wrinkle (*d*) can be written as the following

3 $$\begin{array}{c}{\rm{\Delta }}W=4{\int }_{0}^{W/4}(1-\,\cos \,\theta ){\rm{d}}s\\ \,\,\,\,\,\,\,\,=\,4\sqrt{\frac{D(1-\,\cos \,{\theta }_{0})}{2{\rm{\Gamma }}}}[{\int }_{0}^{{\theta }_{0}}\frac{{\rm{d}}\theta }{\sqrt{\cos \,\theta -\,\cos \,{\theta }_{0}}}-{\int }_{0}^{{\theta }_{0}}\frac{\cos \,\theta d\theta }{\sqrt{\cos \,\theta -\,\cos \,{\theta }_{0}}}]\end{array}$$

4 $$d={\int }_{0}^{W/2}\sin \,\theta {\rm{d}}s=4(1-\,\cos \,{\theta }_{0})\sqrt{\frac{D}{2{\rm{\Gamma }}}}$$

5 $$W-{\rm{\Delta }}W={\int }_{-W/2}^{W/2}\cos \,\theta {\rm{d}}s=4\sqrt{\frac{D(1-\,\cos \,{\theta }_{0})}{2{\rm{\Gamma }}}}{\int }_{0}^{{\theta }_{0}}\frac{\cos \,\theta d\theta }{\sqrt{\cos \,\theta -\,\cos \,{\theta }_{0}}}$$

Heights of graphene wrinkle are restricted by

6 $$0\le d\le 8\sqrt{\frac{D}{2{\rm{\Gamma }}}}$$

Substituting the experimental obtained values of Γ=0.72 J/m^2^ ^21^ and *D* = 0.192 nN·nm^24^, theoretical results can be used to interpret simulation finds. Both theory results based on the Eqs (3)–(6) and simulation results are plotted in Fig. 3b, in general, theoretical results are well consistent with simulation observations. Figure 3b clearly shows that height of graphene wrinkles is nearly linear dependent on compression strain over the entire strain range from *ɛ* = 2.55% to *ɛ* = 71.46%. Theoretical results show that an increase of graphene wrinkle width should only be seen for *ɛ* < 37.54% and the decrease of graphene wrinkles can be seen for *ɛ* > 37.54%. The maximum width of wrinkles is less than 2.6 nm which is large smaller than the experimental observations (30 nm–40 nm).

### Theory analysis with interlayer molecules

The theoretical results can fit and predict the MD results very well, however, neither of them can explain experimental observations, so there must be some other mechanisms need to be seriously considered. It is crucial to understand the details of the growth process. We note that directly grow graphene on copper involving a surface catalyzed process which is generally attributed to the low solubility of carbon (<0.001 at.%) in copper and the surface diffusion of carbon atoms on copper substrate. First expose copper substrate to the mixture of methane and hydrogen gas. Depending upon the temperature, methane pressure, methane flow and hydrogen partial pressure, methane decomposes to form hydrogen and carbon atoms. Finally, aggregation of carbon atoms on Cu surface form graphene nucleation centers and form graphene films consequently.

**Figure 4:**
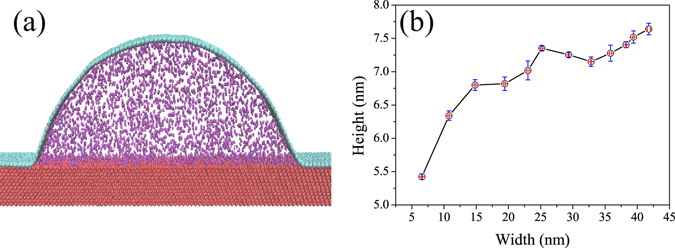
Typical MD simulation results of graphene wrinkle morphology with inter-molecules and the simulation results at temperature *T* = 300 K. (**a**) Typical MD simulation results of graphene wrinkle morphology with inter-molecules. The MD simulation model contains three parts, upper cyan graphene, interlayer hydrogen molecules and lower red copper substrate. (**b**) Simulation results of graphene wrinkles with various number of interlayer hydrogen molecules, the horizontal and vertical axis represents the width and height of graphene wrinkles. The error bars represent the standard deviation of five independent calculations for the same model.

The key factor during the CVD process is that there may be some molecules (mainly hydrogen molecules and hydrocarbon molecules) dissolve in the copper substrate, after cooling to room temperature, precipitation of hydrogen molecules happens. However graphene is impermeable to molecules even hydrogen molecules^27^, so there are a lot of molecules between graphene and copper substrate. In order to involve this effect, we built MD models with various number of interlayer molecules and calculated the final morphology. To be simplified, we only consider hydrogen as the interlayer molecules. Figure 4a shows a typical MD simulation result of graphene morphology with inter-molecules at temperature *T* = 300 K, the calculation model contains three parts, upper cyan graphene, interlayer hydrogen molecules and lower red copper substrate. Morphologies of graphene wrinkles with different numbers of interlayer molecules can be found in Fig. 4b which plots the width of graphene wrinkle versus height. It is clear demonstrated that as the number of inlayer molecules increases, both width and height of wrinkles increases. These theoretical results can be used to interpret the experimental findings. In the simulation range, the height is predicted to be changed from 5.4 nm to 7.6 nm, and besides, the width of wrinkles is predicted to be changed from 6.5 nm to 41.8 nm. This is consistent with the experimental observations (Fig. 1). The interlayer molecules can supply additional pressure that enlarges both width and height of graphene wrinkle.

## Conclusion

In the present work, we report the unexpected morphology of graphene wrinkles on copper substrate. In order to systematically understand mechanisms of the unexpected phenomenon, we performed several MD simulations without interlayer molecules. The calculated widths of wrinkles are largely smaller than the experimental observed. In addition, a theoretical model based on Lagrangian multiplier method was proposed which fits and predicts the simulations results well. However, both the MD results and theory results are contradicted with experimental observed. Based on the above analysis, we performed inter-molecules MD calculations. The obtained results are consistent with the experimental observations. The interlayer molecules can supply additional pressure that enlarges both width and height of graphene wrinkle.

## Electronic supplementary material

Supplementary Information
