# Supplementary material for: Observation of the unexpected morphology of graphene wrinkle on copper substrate
Source: Sci Rep. 2017 Aug 15;7:8244. doi: 10.1038/s41598-017-08159-8 (PMC5557975; doi:10.1038/s41598-017-08159-8)
Supplement: Supplementary file 1 — Supplementary Information [file 41598_2017_8159_MOESM1_ESM.pdf]

Supplementary Information to Observation of the unexpected morphology of graphene wrinkle on copper substrate

Wen Wang[1*], Shudu Yang[2], Ashu Wang[3*]

[1]School of Mechanical Engineering, Southwest Jiaotong University, Chengdu 610031, China

[2]Sichuan Branch of Meteorological Training Institute CMA, Chengdu 610072, China

[3]School of Information Science and Engineering, Chengdu University, Chengdu 610106, China

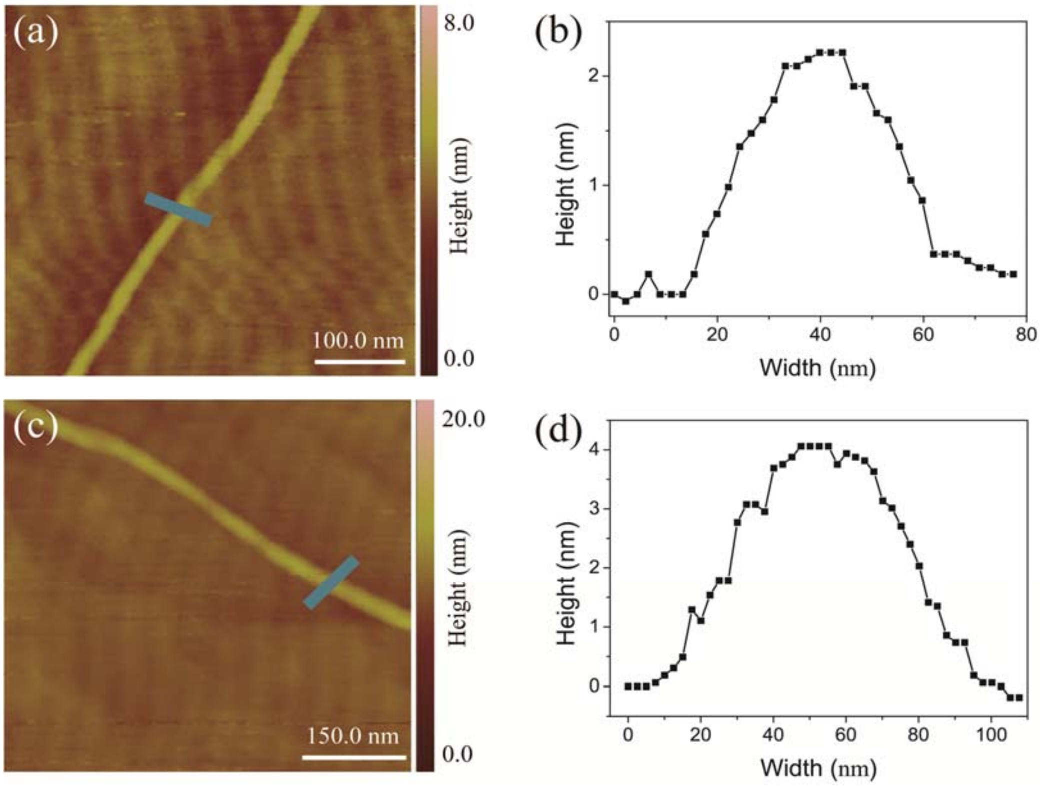

Figure S1. Experimental AFM morphology image of graphene wrinkles on copper sustrate by using conventinal tips with a diameter of 10 nm. (a)&(c) AFM morphology image of graphene wrinkles obtained by CVD methods. (b)&(d) The AFM heights of the cross-sectionl trace of the line marked in Fig. S1a&c respectively.

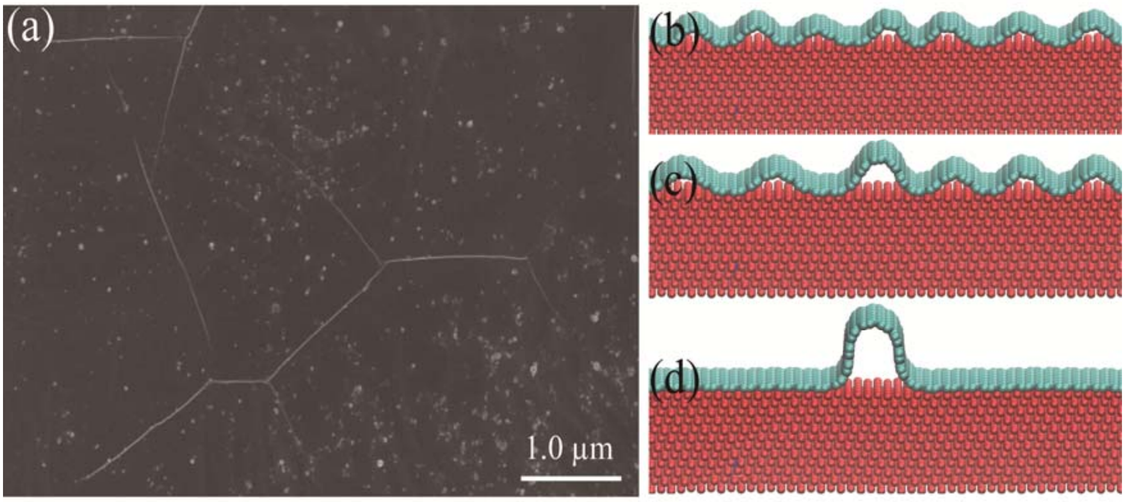

Figure S2. SEM charaterision of graphene wrinkles and the merge of neighbored wrinkles on copper substrate in MD simulations. (a) SEM charaterision of graphene wrinkles, due to the non-uniform distribution of strain and merge of wrinkle, the distribution of graphene wrinkle are not uniform. (b)-(d) The evolution of graphene wrinkles, several neighbored wrinkles merged to forme a larger wrinkle under constant external compression strain.

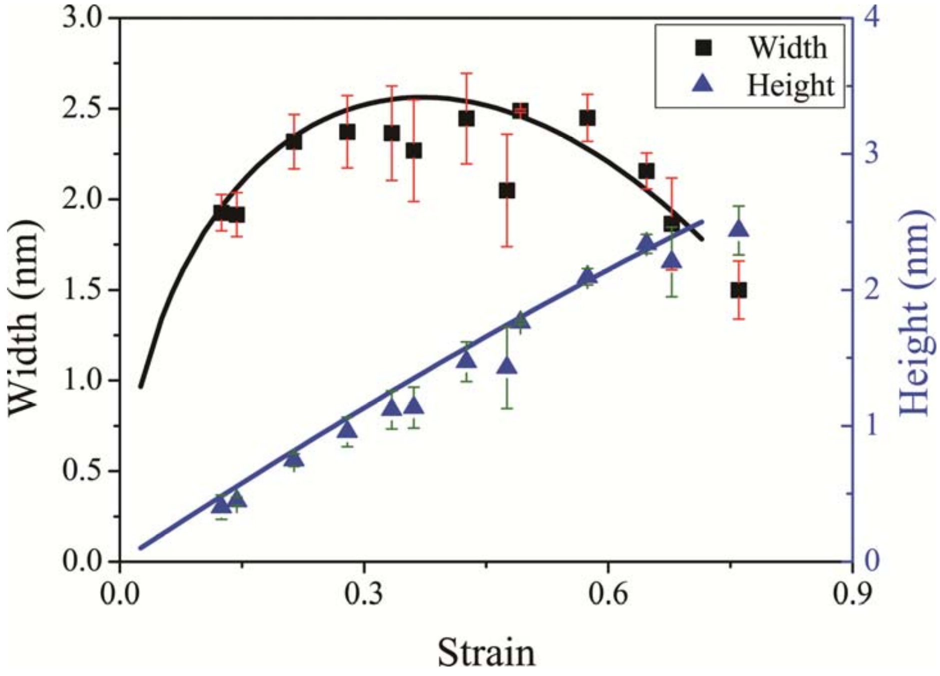

Figure S3. MD simulation results at T = 1 K. For small strain, both h and W increase with strain; for large strain, h still increases with strain, however, W surprisingly decreses with strain. The error bars represent the standard deviation of five independent calculations.
